# Therapeutic Role of Pharmacological Chaperones in Lysosomal Storage Disorders: A Review of the Evidence and Informed Approach to Reclassification

**DOI:** 10.3390/biom13081227

**Published:** 2023-08-07

**Authors:** Ian Keyzor, Simon Shohet, Jeff Castelli, Sheela Sitaraman, Biliana Veleva-Rotse, Jill M. Weimer, Brian Fox, Tobias Willer, Steve Tuske, Louise Crathorne, Klara J. Belzar

**Affiliations:** 1 Amicus Therapeutics Ltd., Marlow SL7 1HZ, UK; 2 Amicus Therapeutics Inc., Princeton, NJ 08542, USA; 3 Amicus Therapeutics Inc., Philadelphia, PA 19104, USA; 4 Prescript Communications Ltd., Letchworth Garden City SG6 3TA, UK

**Keywords:** lysosomal storage disorders, molecular chaperone, Fabry disease, Gaucher disease, Pompe disease, Niemann–Pick disease type C

## Abstract

The treatment landscape for lysosomal storage disorders (LSDs) is rapidly evolving. An increase in the number of preclinical and clinical studies in the last decade has demonstrated that pharmacological chaperones are a feasible alternative to enzyme replacement therapy (ERT) for individuals with LSDs. A systematic search was performed to retrieve and critically assess the evidence from preclinical and clinical applications of pharmacological chaperones in the treatment of LSDs and to elucidate the mechanisms by which they could be effective in clinical practice. Publications were screened according to the Preferred Reporting Items for Systematic reviews and Meta-Analyses (PRISMA) reporting guidelines. Fifty-two articles evaluating 12 small molecules for the treatment of seven LSDs are included in this review. Overall, a substantial amount of preclinical and clinical data support the potential of pharmacological chaperones as treatments for Fabry disease, Gaucher disease, and Pompe disease. Most of the available clinical evidence evaluated migalastat for the treatment of Fabry disease. There was a lack of consistency in the terminology used to describe pharmacological chaperones in the literature. Therefore, the new small molecule chaperone (SMC) classification system is proposed to inform a standardized approach for new, emerging small molecule therapies in LSDs.

## 1. Introduction

Lysosomal storage disorders (LSDs) are a heterogeneous group of rare diseases primarily caused by mutations in genes encoding enzymes responsible for normal lysosomal function [1]. For example, missense mutations in genes encoding lysosomal enzymes may cause them to misfold, leading to endoplasmic reticulum (ER) retention and/or early degradation [2,3]. Partial or complete deficiency of the lysosomal enzyme leads to progressive accumulation of the substrate of the mutant lysosomal enzyme, resulting in additional cell toxicity and death [1]. There are 70 different LSDs, including Gaucher disease, Fabry disease, Pompe disease and Niemann–Pick disease type C (NPC). Gaucher disease is the most common LSD, with a global prevalence of 1.5 cases per 100,000 live births [4]. It occurs due to mutations in the *GBA1* gene encoding glucocerebrosidase (GCase), a lysosomal enzyme that converts D-glucosylceramide into ceramide and D-glucose [5]. *GBA1* mutations commonly result in GCase misfolding, followed by retention within the ER and premature degradation [5].

Fabry disease is caused by the deficient activity of lysosomal glycosidase due to mutations in the *alpha-galactosidase A* (*α-Gal*) gene located on the X-chromosome, which results in the storage of excess cellular glycosphingolipids [6]. In Pompe disease, mutations in the *acid-alpha-glucosidase* (*GAA*) gene cause a deficiency of the lysosomal enzyme GAA, leading to progressive, intralysosomal glycogen accumulation in multiple tissues and organs [7]. NPC types 1 and 2 (NPC1 and NPC2) are enzymes involved in cholesterol efflux from late lysosomal and endosomal compartments [8]. NPC disease typically results from missense mutations (70–80% cases) in the *NPC1* gene, resulting in misfolding and premature degradation of the NPC1 protein, which leads to the progressive onset of neurological symptoms such as loss of motor function and cognitive impairment [8].

The drug development pipeline for LSDs is rapidly evolving worldwide [9], with several different treatment approaches now used in routine clinical practice, including enzyme replacement therapy (ERT), substrate reduction therapy (SRT), and pharmacological chaperone therapy (PCT) (Table 1). ERT is the most established treatment approach for LSDs resulting from impaired lysosomal enzymes [10]. This approach involves the recurrent administration of exogenous recombinant protein that replaces the specific defective enzyme in order to reduce substrate accumulation [10]. At least 15 ERT products have been developed and approved in Europe for nine different LSDs (Gaucher, Fabry, and Pompe disease, ceroid lipofuscinosis type 2 disease, as well as five mucopolysaccharidoses (MPS I, MPS II, MPS VIA, MPSVI, and MPSVII)) [11]. Although ERT is a highly effective treatment for many individuals with LSDs, it remains a less than favorable therapeutic option for others, e.g., those hypersensitive to the recombinant protein, with mutations in non-lysosomal enzymes, or with LSDs that affect tissues and organs less accessible by intravenous delivery of replacement protein [12,13]). Since ERT can lead to infusion-associated reactions and the formation of neutralizing antidrug antibodies that reduce the efficacy of therapy for some individuals, alternative treatment approaches are warranted [12,13]. Regular ERT infusions can also be inconvenient and have a major impact on a person’s home and work life [14]. Furthermore, some individuals with Pompe disease and who are cross-reactive immunologic material-negative have a poor clinical response to ERT secondary to high sustained antibody titers [15]. Over the past 50 years, there has been an increased focus on the therapeutic role of small molecules, with a remarkable 124 orphan drug designations granted in the US alone for compounds intended to treat 28 LSDs [9]. Within the small molecule development space, SRT and PCT have been shown to provide benefit for the treatment of some LSDs. As the name suggests, rather than directly restoring the activity of the defective enzyme, SRT aims to attenuate the biosynthesis of the accumulated substrate. One SRT drug, N-butyl-deoxynojirimycin (NB-DNJ/miglustat; Zavesca^®^; Actelion Pharmaceuticals US Inc, San Francisco, CA, USA), is approved in Europe for mild to moderate type 1 Gaucher disease in individuals for whom ERT is unsuitable and for the treatment of progressive neurological manifestations for individuals with NPC disease (approved by the European Medicines Agency (EMA) in 2002 and 2009, respectively) [11]. Miglustat can also act either as a competitive inhibitor of glucosylceramide synthase to decrease the synthesis and accumulation of glucosylceramide in Gaucher disease or as an enzyme stabilizer in Pompe disease [11]. Another SRT, eliglustat (Cerdelga^®^; Sanofi, Paris, France), was approved by the EMA in 2015 as a first-line treatment for Gaucher disease type 1 [11].

The term “pharmacological chaperone therapy” or “PCT” was first coined in 2000 to describe the category of exogenously administered small molecules that restore folding and trafficking defects of misfolded proteins in LSDs. The EMA approved the first commercially available PCT, migalastat (Galafold^®^; Amicus Therapeutics Inc., Philadelphia, PA, USA), in 2016, for long-term treatment of adults with Fabry disease who have an amenable mutation (i.e., a mutation that is responsive to treatment) [11]. Migalastat binds and stabilizes endogenous mutant α-Gal enzyme, thereby increasing its activity relative to the specific amenable mutation and improving trafficking to the lysosome, i.e., it reduces ER retention of mutant endogenous α-Gal. Most pharmacological chaperones, such as migalastat, have been reported to bind to the active site of their target enzyme, acting as competitive inhibitors [16]. Second-generation pharmacological chaperones that bind to allosteric sites to stabilize and protect mutant enzymes from degradation without interfering with their activity may also have therapeutic potential [17]. Studies have shown that PCT enables the conformational stabilization of the target protein by promoting more favorable free energy states than the unbound state at neutral pH [18]. Once in the acidic environment of the lysosomes, the pharmacological chaperone dissociates from the mutant enzyme, thus restoring some of its residual catalytic activity [19].

The clinical utility of PCTs to improve the stability of exogenous ERT has also been investigated in Pompe disease, including the use of miglustat in combination with alglucosidase alfa, the first approved recombinant human GAA (rhGAA) for Pompe disease [20], and cipaglucosidase alfa [21], a next-generation rhGAA with cellularly derived bis-phosphorylated N-glycans to improve its cellular uptake through cation-independent mannose-6-phosphate receptors while retaining its capacity to be fully processed into the most active form of the enzyme [21]. In a proof-of-concept study, miglustat increased and prolonged GAA enzyme activity in dried blood spots (DBS) when compared with alglucosidase alfa alone, which the authors concluded is suggestive of a stabilization effect of miglustat on alglucosidase alfa in plasma, as was demonstrated in preclinical studies [20]. Furthermore, building on preclinical data demonstrating that miglustat could bind to, stabilize, and minimize the inactivation of cipaglucosidase alfa [20,22,23,24,25], the safety and effectiveness of cipaglucosidase alfa plus miglustat in adults with Pompe disease was evaluated in the randomized, phase III PROPEL study (NCT03729362) [21]. The two-component miglustat plus cipaglucosidase alfa therapy for Pompe disease was recently approved by the European Commission for treating adults with late-onset Pompe disease (LOPD) and is currently under regulatory review by the US Food and Drug Administration (FDA) [26].

Protein folding and degradation are naturally regulated by endogenous molecules known as molecular chaperones (e.g., the heat shock protein 70 (HSP70) superfamily), which, together with the ubiquitin–proteasome system and autophagy, are central components of protein quality control [27]. Proteostasis regulators (PRs) have been shown to enhance the expression and functions of endogenous molecular chaperones and regulators of the endoplasmic reticulum quality control system to facilitate protein folding and minimize misfolding in vivo [28]. PRs offer an alternative treatment approach for many human diseases associated with altered protein conformation, including LSDs [28]. Arimoclomol, for example, is an orally available small molecule PR that amplifies the heat shock response and production of heat shock proteins to prevent protein misfolding and activate lysosomal function. The efficacy and safety of arimoclomol to target protein misfolding and improve lysosomal function in individuals with NPC is currently being evaluated in a phase II/III clinical trial (clinicaltrials.gov identifier: NCT02612129) [8].

Due to the rapid expansion of various types of small molecule therapies with different mechanisms of action under investigation for LSDs, we conducted a systematic review to examine the available evidence from the literature for preclinical and clinical applications of pharmacological chaperones in the treatment of LSDs. Based on our findings, we propose a new classification for small molecules in the treatment of LSDs that considers differences in therapeutic approach and mechanism of action.

**Table 1:** Different therapeutic approaches for treating LSDs and current descriptions of approved therapies (in at least one geography).

	Enzyme Replacement Therapy (ERT)	Substrate Reduction Therapy (SRT)	Pharmacological Chaperone Therapy (PCT)
**Molecular or physiological target**	Absent or reduced protein function	Metabolic cascade	Endogenous and/or exogenous protein trafficking/stability
**Mechanism of action**	Substitute or addition of missing or deficient endogenous enzyme with exogenously delivered enzyme	Interferes with the abnormalaccumulation of substrate	Stabilize and restore intracellular trafficking to increase activity of endogenous mutant enzymes Stabilize exogenous ERTs during trafficking from blood to site of action (e.g., lysosome)
**Approved therapy**	Agalsidase alfa and beta for Fabry disease; alglucosidase alfa, avalglucosidase alfa and cipaglucosidase * for Pompe disease; and velaglucerase and imiglucerase for Gaucher disease [11]	Eliglustat [29] for Gaucher disease and miglustat [30] for Gaucher disease and NPC disease.	Example: migalastat for Fabry disease [31,32,33,34,35,36,37,38,39,40,41] Example: miglustat * for Pompe disease [26]

* Cipaglucosidase alfa is approved in Europe with the enzyme stabilizer miglustat as a two-component therapy for Pompe disease. Abbreviations: LSDs, lysosomal storage disorders.

## 2. Materials and Methods

A systematic review was conducted following the general principles published by the UK NHS Centre for Reviews and Dissemination (CRD) and is reported as per the Preferred Reporting Items for Systematic reviews and Meta-Analyses (PRISMA) reporting guidelines [42]. PubMed^®^ and Embase^®^ via Ovid^®^ databases were searched using the following strategy: terms for pharmacological chaperones utilizing a combination of free-text terms (i.e., searching in the title/abstract) and subject headings (e.g., MeSH in Medline) and standardized study design filters. Each search was adapted using the appropriate syntax for each database platform (Table S1). All databases were searched from inception to 20 February 2023. No date or language restrictions were applied. The search strategies for both databases are available in the supplementary material (Table S1). All search results were imported into reference management software (Covidence https://www.covidence.org/, accessed on 1 July 2023) and de-duplicated using both EndNote’s de-duplication features and manual checks.

### 2.1. Study Selection

The inclusion criteria were: (a) research papers and reports where pharmacological chaperones in people with LSDs were the main research topic for the study; (b) only papers written in English or where an English language version was available; and (c) study designs including randomized or non-randomized trials, and economic evaluations. Studies were excluded for the following reasons: abstract only, commentary or non-systematic review articles, letters, and editorials. An initial sample of 10% of abstracts (n = 253) was screened independently by two reviewers to pilot the inclusion criteria and ensure consistency prior to undertaking title and abstract screening (inter-rater agreement was 96.4% and discrepancies were resolved by discussion). Duplicate studies were removed. The remaining studies (n = 2877) were single screened. Where the title or abstract met the criteria (or if this was unclear), the full text was retrieved and screened. Full-text screening was undertaken by two reviewers (the inter-rater agreement was 94.2%).

### 2.2. Data Extraction Process and Included Studies

**Figure 1:**
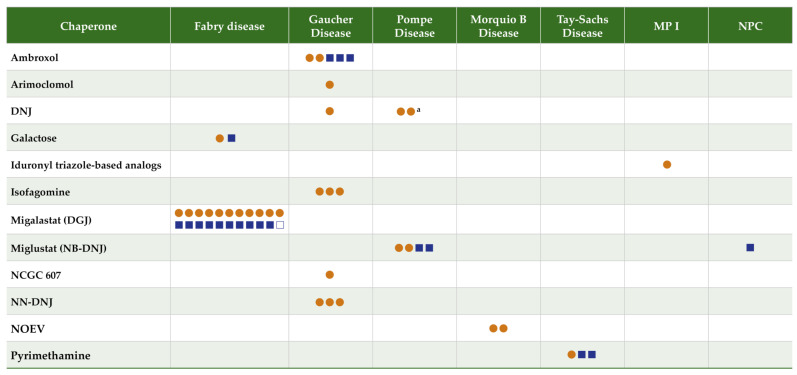
Literature evidence map for chaperones in lysosomal storage disorders. Orange circles = preclinical studies. Blue squares = clinical studies. Closed squares = one study; open square = 2 articles reporting data from the same study. ^a^ One citation reported an analysis including both chaperones N-butyl-deoxynojirimycin (NB-DNJ) and 1-deoxynojirimycin (DNJ), counted twice. Abbreviations: DGJ, 1-deoxygalactonojirimycin/migalastat; DNJ, 1-deoxynojirimycin; NB-DNJ, N-butyl-deoxynojirimycin/miglustat; NN-DNJ, N-Nonyldeoxynojirimycin; NOEV, N-octyl-4-epi-beta-valienamine; NPC, Niemann–Pick Disease Type C; MPSI, Muco-polysaccharidosis 1.

Data were extracted by one reviewer and checked by a second using a standardized form that included information on the disease, chaperone, deficient enzyme, mechanism of action, study type, study objective, and summary findings. A total of 52 articles were identified for inclusion (refer to the PRISMA study selection flow diagram; Supplementary Material, Figure S1). Figure 1 summarizes the literature evidence by LSD type and evidence type for the identified chaperones.

## 3. Results

### 3.1. Literature Evidence

Fifty-two articles evaluating 12 chaperones (1-DGJ/migalastat; galactose; isofagomine; arimoclomol; N-Nonyldeoxynojirimycin (NN-DNJ); ambroxol; NCGC607; NB-DNJ/miglustat; 1-DNJ/AT2220/duvoglustat; NOEV; pyrimethamine; iduronyl triazole-based analogs) for the treatment of seven LSDs are included in this review.

#### 3.1.1. Fabry Disease

A total of 25 articles (13 clinical and 12 preclinical studies) were identified evaluating chaperones in Fabry disease, including galactose (two articles; one preclinical and one clinical study) and migalastat (24 articles; 11 preclinical and 12 clinical studies). The split between preclinical and clinical studies was equal. A summary of the included clinical studies for Fabry disease is shown in Table 2 [31,32,33,34,35,36,37,38,39,40,43,44,45]. A summary of the preclinical studies is shown in the Supplementary Material (Table S2) [46,47,48,49,50,51,52,53,54,55,56,57].

#### 3.1.2. Gaucher Disease

A total of 14 articles (three clinical studies) were identified evaluating six chaperones in Gaucher disease: isofagomine (three preclinical articles), arimoclomol (one preclinical article), 1-deoxynojirimycin (DNJ) (one preclinical article), ambroxol (five articles; two preclinical and three clinical studies), NN-DNJ (three preclinical articles), NCGC607 (one preclinical article). Most of the articles were preclinical studies (78.6%). A summary of the included clinical studies for Gaucher disease is shown in Table 3 [58,59,60]. A summary of the preclinical studies is shown in the Supplementary Material (Table S3) [61,62,63,64,65,66,67,68,69,70,71].

#### 3.1.3. Pompe Disease

A total of six articles (two clinical studies) were identified evaluating chaperones in Pompe disease: NB-DNJ/miglustat (two preclinical articles), DNJ (two preclinical articles; note one article included both NB-DNJ and 1-DNJ), NB-DNJ/miglustat plus ERT (one clinical article), and miglustat plus cipaglucosidase alfa combination (one clinical article). The majority of the articles were preclinical studies. A summary of the included clinical studies for Pompe disease is shown in Table 4 [20,21]. A summary of the preclinical studies is shown in the Supplementary Material (Table S4) [22,72,73,74].

#### 3.1.4. GM1 Gangliosidosis—Morquio B Disease

A total of two articles (no clinical studies) were identified evaluating chaperones in Morquio B disease: N-octyl-4-epi-beta-valienamine (NOEV). A summary of the preclinical studies is shown in the Supplementary Material (Table S5) [75,76].

#### 3.1.5. GM2 Gangliosidosis—Tay–Sachs Disease

A total of three articles (one preclinical study and two clinical studies) were identified evaluating chaperones in Tay–Sachs disease: pyrimethamine (three articles). A summary of the included clinical studies for Tay–Sachs disease is shown in Table 5 [77,78]. A summary of the preclinical studies is shown in the Supplementary Material (Table S6) [79].

#### 3.1.6. Mucopolysaccharidosis I

One preclinical article was identified evaluating pharmacological chaperones in mucopolysaccharidosis I disease (iduronyl triazole-based analogs). A summary of the preclinical study is shown in the Supplementary Material (Table S7) [80].

#### 3.1.7. NPC

One clinical article was identified evaluating pharmacological chaperones in NPC: miglustat. A summary of the included clinical study for NPC disease is shown in Table 6 [81].

### 3.2. Mechanism of Action and Rationale for Reclassification of Small Molecule Chaperones

The mode of action (MoA) for individual small molecule therapies analyzed in this paper varies and the exact MoA remains to be fully elucidated for many. In Fabry disease, migalastat reversibly binds to the active site of amenable mutations of endogenous agalactosidase and stabilizes the mutant enzyme to facilitate trafficking to the lysosome [16]. Migalastat is described in the literature as having multiple modes of action, including molecular binding and rescue of protein folding, stabilization and ‘’active’’ trafficking, i.e., it ‘‘accompanies’’ the deficient endogenous α-Gal protein as it is transported from the ER through the trans-Golgi network to the lysosomes [16]. In preclinical studies, the expectorant ambroxol showed pH-dependent affinity for the lysosomal hydrolase GCase with decreasing inhibition at lysosomal pH. The exact mode of action of ambroxol in Gaucher disease is unclear [82]. More recently, Pantoom et al. (2022) showed that ambroxol has little in vitro ability to increase the specific activity of GCase [83,84]. Further studies are required to elucidate whether ambroxol plays a regulatory role in the proteostasis network [83,84].

Some small molecules may exert a different MoA in different LSDs. Miglustat acts as a competitive, reversible glucosylceramide synthase inhibitor and has been approved as an SRT in Gaucher disease and NPC [11]. However, in Pompe disease, miglustat, in combination with ERT, has demonstrated potential as a stabilizer of exogenously administered recombinant GAA [20]. Our literature review also identified differences in how chaperones are described and classified. For example, Parenti et al. (2015) classify pharmacological chaperones as small molecule ligands that selectively bind, stabilize, and traffic unstable proteins [16]. However, Frustaci et al. (2001) evaluated the role of exogenously administered galactose infusion therapy for the treatment of an individual with Fabry disease, describing galactose as a ‘‘chemical chaperone’’ able to bind reversibly, stabilize, and assist in the trafficking of endogenous mutant α-Gal from the ER to the lysosome [43]. Notably, the terms chemical or molecular chaperone are typically misapplied in the literature, e.g., these terms have been used to describe endogenous protein chaperones such as HSP70 rather than an exogenously administered pharmacological chaperone that binds to deficient endogenous α-Gal. Abian et al. (2011) used the term ‘’chemical chaperones’’ for small molecules unable to leave the ER [85]. In contrast, Okumiya et al. (2007) described miglustat as a ‘’chemical chaperone’’ that promotes export from the ER to the lysosomes and stabilizes the activity of endogenous mutant GAA species in individuals with Pompe disease [72].

**Figure 2:**
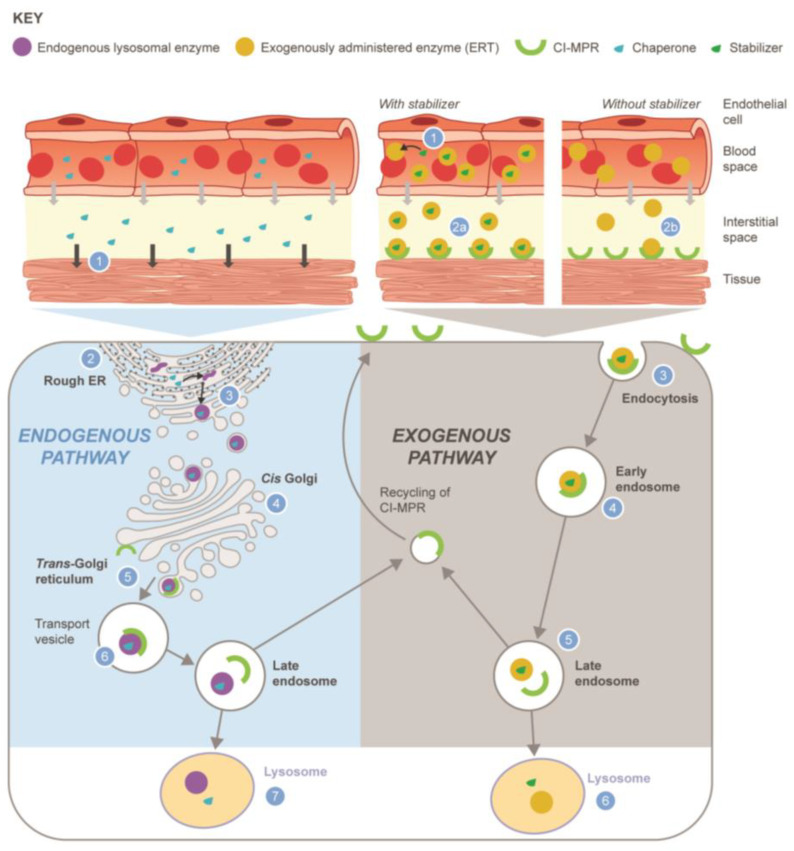
Reclassification of small molecule chaperones (SMC) into two distinct pathways: monotherapy chaperone (**left panel**) or combined/co-administered stabilizer (**right panel**). Chaperone/endogenous lysosomal enzyme (**left**): (1) Chaperone leaves circulation by paracellular diffusion, enters the cell, and distributes to the ER within target tissues; (2) synthesis of unstable endogenous mutant enzyme protein on the rough ER; (3) chaperone binds and stabilizes endogenous enzyme by facilitating the correct folding required to progress from the ER to the Golgi; (4) enzyme–chaperone complex transported through Golgi; (5) enzyme–chaperone complex phosphorylated and binds to CI-MPR; (6) enzyme–chaperone complex transported through endosomes to lysosome; (7) chaperone dissociates from endogenous enzyme due to low pH and high substrate concentration in the lysosome, resulting in endogenous enzyme being available within lysosome. Stabilizer/exogenously administered lysosomal enzyme (**right**): (1) Stabilizer binds to exogenously administered enzyme in the blood; (2a) enzyme–stabilizer complex binds to CI-MPR; (2b) without the stabilizer, less active enzyme is available to bind to the CI-MPR; (3) enzyme–stabilizer complex undergoes endocytosis into the muscle cell after binding CI-MPR; (4) enzyme–stabilizer complex transported through endosomes; (5) enzyme–stabilizer complex transported to lysosome; (6) stabilizer dissociates from exogenous enzyme in the lysosome. Abbreviations: CI-MPR, cation-independent mannose 6-phosphate receptor; ER, endoplasmic reticulum; ERT, enzyme replacement therapy.

Differences in the MoA of small molecule chaperones identified in the literature and the terminology used to describe them vary significantly and are often contradictory. Moreover, the current term ‘’pharmacological chaperone’’ is inadequate to fully differentiate between the emerging small molecule therapies for LSDs. Therefore, we propose a reclassification to standardize terminology based on the MoA of the small molecule therapy in treating LSDs (Table 7 and Figure 2). The SMC classification system is a simple tool to better describe and categorize existing and future small molecule pharmacological chaperones approved to treat LSDs by type of therapy (i.e., monotherapy or combination), type of action (i.e., chaperone or stabilizer), target enzyme (i.e., exogenous ERT or endogenous mutant), and site of action/trafficking (i.e., intracellular or circulation). The differences between the two proposed SMC groups are depicted in Figure 2. Notably, the SMC classification currently includes two distinct groups and may need to be further revisited as novel MoAs are validated.

## 4. Discussion

This review presents the available literature evidence in relation to the therapeutic role of small molecule chaperones in LSDs. The clinical evidence suggests that small molecule chaperones can be effective and well tolerated. Most of the clinical evidence identified evaluated migalastat for the treatment of Fabry disease. This was expected since the first studies using pharmacological chaperones were conducted for Fabry disease, and migalastat was the first pharmacological chaperone approved for treating individuals with Fabry disease and amenable mutations [11]. Our literature review highlighted a lack of consistency in terminology in articles that describe pharmacological chaperones, which could be attributable to evolution in their development and varying and often unclear MoAs. Therefore, we propose a new classification system to inform a standardized approach, which we have referred to as the SMC classification system.

Preclinical evidence suggested pharmacological chaperones could ‘‘theoretically’’ enhance enzyme activity for all identified LSDs. However, while some mutations for LSDs could respond to small molecule chaperone therapy, amenability will likely continue to be a limiting factor. The systematic literature review identified a wealth of active preclinical research in Fabry disease (n = 12 studies) and Gaucher disease (n = 11 studies). The concept for chaperone-stabilized recombinant enzyme therapy has been explored in preclinical models of Pompe disease. Coadministration of miglustat with cipaglucosidase alfa has been shown to improve rhGAA activity compared to administration of cipaglucosidase alfa alone in GAA knockout mice [22,23]. Miglustat was also shown to increase the circulation half-life of cipaglucosidase alfa in mouse, rat, and monkey models of Pompe disease [25]. When miglustat was administered with cipaglucosidase alfa, the two-component treatment reversed or significantly improved all aspects of the Pompe disease pathogenesis in non-symptomatic GAA-deficient mice with fully developed muscle pathology [25]. These studies suggest that cipaglucosidase alfa plus miglustat can reverse not only the primary defect of Pompe disease, lysosomal glycogen accumulation, but also secondary events resulting from lysosomal dysfunction in the muscle of GAA knockout mice [20,22,23,24,25]. Fewer preclinical studies were identified for rarer LSDs (Pompe disease, n = 4; Morquio B disease, n = 2; Tay–Sachs disease, n = 1; mucopolysaccharidosis I, n = 1; and NPC, n = 0).

The literature identified that chaperone treatment was effective in individuals with Fabry disease with a wide range of genotypes, phenotypes, and disease severity, including those with amenable mutations with multiple organ system involvement at baseline [36,39]. In Fabry disease, chaperone therapy reduced clinical deterioration (i.e., renal, cardiac and neurologic function, pain symptoms, and health status were unchanged), suggesting those on chaperone therapy maintained disease stability [31]. The long-term benefit of chaperone treatment has been demonstrated through maintenance of renal function over ≤8.6 years, irrespective of treatment status, sex, and phenotype in individuals with Fabry disease and amenable mutations [40].

In Pompe disease, the use of miglustat as a stabilizer of rhGAA has been reported, first for alglucosidase alfa [20] and subsequently for cipaglucosidase alfa [21]. The evaluation of miglustat in combination with cipaglucosidase alfa is particularly relevant since this two-component therapy has now received regulatory approval for the treatment of adults with LOPD disease by the EMA and is currently under investigation by the US FDA [26]. Miglustat has been reported to minimize inactivation of cipaglucosidase alfa in the circulation, making more targeted enzyme available to target tissue. A phase III clinical study demonstrated meaningful improvements in musculoskeletal and respiratory endpoints for individuals switching from alglucosidase alfa to cipaglucosidase alfa plus miglustat [21].

Overall, although the clinical studies identified herein show chaperones and/or stabilizers are promising therapeutic tools in LSDs, their success and clinical application are currently limited for most LSDs, except for Fabry disease, and potentially in the future for Pompe disease.

The strengths of this literature review are that the searches were systematic, and two authors reviewed each article. This enhances and adds robustness to the non-systematic approach previously reported in the review published by Ligouri et al. (2020) [86] because systematic reviews typically use more comprehensive search strategies that reduce biases [87]. However, the full text of a few articles could not be accessed, and studies not written in the English language that may have also been relevant to the findings in our literature review were excluded. Additionally, in LSDs, the mechanism of action of some small molecule entities can vary by disease type, e.g., miglustat acts as a substrate reduction therapy in Gaucher disease and as an enzyme stabilizer in Pompe disease. Furthermore, given the specified research question, a quality assessment of the included studies was not conducted. An additional strength is that our results update previous reviews and move the pharmacological chaperone discussion forward by proposing a new classification for emerging small molecule therapies in LSDs.

## 5. Conclusions

A substantial amount of preclinical data support the potential of small molecule chaperones/stabilizers as treatments for Fabry disease, Gaucher disease, and Pompe disease. However, additional preclinical studies are required for other LSDs, including Morquio B disease, Tay–Sachs disease, mucopolysaccharidosis I, and NPC, to elucidate the mechanisms by which small molecule chaperones/stabilizers could be effective in clinical practice. Most clinical studies identified in this review were with migalastat for Fabry disease, which demonstrated a beneficial effect with respect to disease outcomes.

With approval of the first oral small molecule chaperone migalastat (Galafold^®^) in Fabry disease, using small molecules to treat other lysosomal storage disorders is a rational approach to explore. Accompanying exogenously administered ERTs with a chaperone/stabilizer, as in the case of cipaglucosidase alfa with miglustat, is another approach that has potential clinical validity in treating LSD individuals. Our study identified an unmet need to understand better in the future the biological pathways that lead to extreme variability in the phenotypes of LSD. Furthermore, our proposed reclassification of small molecule chaperone therapies based on their MoAs will help standardize the terminology of these molecules in clinical development, which will subsequently help researchers, clinicians, and drug developers to focus on and rationally apply these promising approaches in the treatment of people with LSDs.

## Figures and Tables

**Table 2:** Clinical evidence summary: Fabry disease.

Author Year (Reference)	Enzyme	MoA (as Per Author’s Comments)	Clinical Study Type	Study Objective	Summary Outcome
**Galactose**					
Frustaci 2001 [43]	α-Gal A	Reversible competitive inhibition to enhance the activity and stability of mutant α-Gal A by binding to the active site and promoting folding, dimerization, and processing, thereby preventing proteasomal degradation.	Case study	Cardiac variant of Fabry disease who had residual α-GAL A activity	For individuals with the cardiac variant whose residual α-Gal A activity can be enhanced in vitro, chaperone-mediated therapy with galactose or other inhibitors may prove safe and therapeutically effective.
**1-deoxygalactonojirimycin (DGJ)/migalastat**					
Giugliani 2013 [37]	α-Gal A	Migalastat HCl targets α-Gal A mutants that maintain catalytic competence. The chaperone competitively binds to the enzyme, resulting in the trafficking of the abnormal α-Gal A mutants to lysosomes and increasing the activity of the enzyme to process GL-3 (Gb3).	Phase II, open-label, uncontrolled study of 12 weeks with extension to 48 weeks	Effect on safety, tolerability, pharmacodynamics, and pharmacokinetics in females with Fabry disease	Migalastat HCl provides a potential novel genotype-specific treatment for FD. It was generally well tolerated. Participants with amenable mutations seem to demonstrate greater pharmacodynamic response to migalastat HCl compared to those with non-amenable mutations. Treatment resulted in GL-3 (Gb3) substrate decrease in females with amenable α-GAL A mutations.
Germain 2016 [39]	α-Gal A	Migalastat, an oral pharmacologic chaperone, stabilizes specific mutant forms of α-Gal A, increasing enzyme trafficking to lysosomes. The stabilization of suitable mutant forms of α-Gal A by migalastat is hypothesized to increase enzyme levels more consistently than enzyme replacement therapy given every 2 weeks.	Phase III, randomized controlled trial comparing migalastat vs. placebo (stage 1) and migalastat open-label (stage 2) (67 participants)	Efficacy and safety in males and females	Participants had a decrease of 50% or more in GL-3 at six months (stage 1), which did not differ significantly between the migalastat group and the placebo group. Participants with suitable mutant α-Gal A who received migalastat for up to 24 months (stage 2) showed reductions in GFR and LVMI. There were no discontinuations due to adverse events related to migalastat; no participants progressed to end-stage renal disease, had strokes, or died from cardiac causes during the study.
Hughes 2017 (corrigendum anon 2018) [36]	α-Gal A	Migalastat stabilizes specific mutant (amenable) forms of α-Gal A by reversibly binding to the active site of the enzyme to promote normal lysosomal trafficking.	Phase III, open-label study comparing migalastat with ERT (ATTRACT, AT1001-012) (57 participants)	Effect on renal function, health, disease substrate, and patient-reported outcomes	Treatment with migalastat was associated with a statistically significant decrease in LVMI and a stabilizing effect on renal function. Migalastat is safe and well tolerated and offers promise as a first-in-class oral monotherapy alternative treatment to intravenous ERT for individuals with Fabry disease and amenable mutations.
Germain 2019 [38]	α-Gal A	Migalastat binds to and stabilizes amenable mutant forms of α-Gal A (‘‘classic phenotype’’), facilitating lysosomal trafficking and increasing lysosomal enzyme activity.	Phase III, randomized controlled trial (FACETS) comparing migalastat with placebo (subgroup 1:14 participants; and subgroup 2:36 participants)	Efficacy and safety	Migalastat benefited males with the classic phenotype, increasing endogenous α-Gal A activity, stabilizing eGFR, reducing LVMI, improving diarrhea symptoms, and reducing PTC GL-3 inclusions and plasma lyso-Gb3 levels.
Lamari 2019 [35]	α-Gal A	The administration of migalastat in people with Fabry disease and amenable mutations improves or stabilizes organ damage, increases α-Gal A activity, and reduces lyso-Gb3 plasma levels.	Retrospective analysis of Fabry males with p.Asn215Ser (N215S, 2 participants)	Effect on α-Gal A activity	The study confirms in vivo the effects of migalastat observed in N215S carriers in vitro. The increase in α-Gal A activity (5.6- and 5.8-fold for the two participants) may be the strongest marker for biochemical efficacy. The normalization of enzyme activity could become the new therapeutic target to achieve.
Muntze 2019 [32]	α-Gal A	Migalastat binds to the active site and stabilizes α-Gal A, which improves substrate catabolism.	Prospective, single-center study after 12 months of migalastat (14 participants)	Effect on efficacy and biomarker changes after 12 months of migalastat treatment	Migalastat therapy led to a rapid, persistent 3-fold median increase in α-Gal A activity, a decrease in lyso-Gb3 levels, and a significant reduction in myocardial mass in males and females with FD that carried amenable mutations.
Lenders 2020; Lenders 2021 [33,34]	α-Gal A	Migalastat stabilizes endogenous α-Gal A and supports a better protein folding in the ER, leading to increased α-Gal A activity, decreased Gb3 accumulation, and increased stability in the lysosomes of those carrying an amenable mutation.	Prospective, observational study (FAMOUS) (59 participants)	Effect on renal, cardiovascular, patient-reported outcomes, and safety at 12 and 24 months	Therapy of previously ERT-treated and untreated people with FD with migalastat for 24 months under ‘’real-world’’ conditions is generally safe and results in a significant 2.3-fold decrease in LVMI from baseline and a moderated renal decline (eGFR). Notably, LVMI decrease was observed in both ERT-experienced and ERT-naïve individuals, both males and females, particularly in those with left ventricular hypertrophy at baseline. Migalastat offers a good treatment alternative in those with FD and amenable mutations, but the treating physician has to monitor the clinical response on a regular basis. The authors found a significant effect on eGFR for the type of antihypertensive used, so it is important to account for antihypertensive use when making clinical decisions.
Bichet 2021 [40]	α-Gal A	As a molecular chaperone, migalastat binds to and stabilizes amenable mutant forms of α-Gal A in the ER, facilitating trafficking of α-Gal A to lysosomes and restoring endogenous enzyme activity.	Post hoc analyses (phase III + OLE) (78 participants)	Effect on long-term renal outcomes	Individuals with Fabry disease and amenable α-GAL A variants had stable renal function during long-term migalastat treatment (≤8.6 years) irrespective of ERT treatment status, sex, or phenotype. Early treatment should be encouraged to stabilize or slow the decline in renal function in people with Fabry disease.
Riccio 2020 [31]	α-Gal A	Migalastat reversibly binds to the active site and stabilizes specific mutant forms of α-Gal A, defined “amenable” to migalastat, promoting trafficking to lysosomes, where it allows the enzyme to catabolize accumulated substrates.	Single-center, observational study (7 participants)	Effects of switch from ERT to migalastat on renal, cardiac, and neurologic function, health status, pain, lyso-Gb3 activity, α-Gal A activity, adverse effects	Switching from ERT to migalastat led to statistically significant increases in α-Gal A activity, reduction in lyso-Gb3 levels and LVMI. Renal, cardiac and neurologic function, pain symptoms and health status were unchanged, suggesting participants maintained disease stability. The frequency of AEs under ERT and migalastat were comparable, concluding that migalastat is valid, safe and well tolerated.
Muntze 2023 [44]	α-Gal A	Not reported	Prospective, multicenter study (37 participants)	‘Medication adherence questionnaire (MAQ)’, ‘SF-36′ and ‘Fabry pain questionnaire’ over a follow-up period of 24 months	Over 24 months, significant improvement of pain and life role limitations due to physical activity was reported (pain: change from baseline: 8.57 points, 95%-CI: 1.32–15.82, *p* = 0.022; role limitations physical: change from baseline: 13.39 points, 95%-CI: 0.61–23.2, *p* = 0.048). Migalastat therapy adherence in FD participants was high and remained high over a follow-up period of 2 years. Patient-reported quality of life remained mostly stable, while pain and physical limitations improved over time.
Camporeale 2023 [45]	α-Gal A	Not reported	Prospective, observational, single-center study (16 participants)	Comprehensive cardiological evaluation before and after 18 months treatment with migalastat in treatment-naïve individuals with genetically confirmed FD and evidence of cardiac involvement	In treatment-naïve individuals with Fabry disease with cardiac involvement, 18-month treatment with migalastat stabilized left ventricular mass and was associated with a trend towards an improvement in exercise tolerance. A tendency to T1 increase was detected by cardiac magnetic resonance. The subset of participants who had significant benefits from the treatment showed an earlier cardiac disease compared to the others.

Abbreviations: α-Gal A, α-Galactosidase A; AE, adverse event; CI, confidence interval; eGFR, estimated glomerular filtration rate; ER, endoplasmic reticulum; ERT, enzyme replacement therapy; Gb3/GL3, glycosphingolipid globotriaosylceramide; FD, Fabry disease; HCL, hydrochloride; lyso-Gb3, globotriaosylsphingosine; LVMI, left ventricular mass index; MoA, mode of action; OLE, open-label extension; PTC, kidney peritubular capillary; T1, longitudinal (spin-lattice) relaxation time.

**Table 3:** Clinical evidence summary: Gaucher disease.

Author Year (Reference)	Enzyme	MoA (as Per Author’s Comments)	Clinical Study Type	Study Objective	Summary Outcomes
**Ambroxol**					
Zimran 2013 [59]	β-Glucosidase	Not reported	Pilot study, off-label use of ambroxol (12 participants)	Tolerability and efficacy	No participant experienced clinically relevant deterioration in disease parameters measured. One participant achieved a robust response relative to baseline: +16.2% hemoglobin; +32.9% platelets; −2.8% liver volume; and −14.4% spleen volume. Three participants, including the participant above, elected to continue on ambroxol for a further 6 months: hemoglobin levels and liver volumes were relatively stable, but platelet counts further increased in the above participant (+52.6% from baseline), and spleen volumes decreased further in all three participants (−6.4%, −18.6%, and −23.4% from baseline).
Narita 2016 [58]	β-Glucosidase	Not reported	Open-label pilot study (5 participants)	Safety, biochemical efficacy, neurological efficacy	High-dose oral ambroxol had good safety and tolerability, significantly increased lymphocyte glucocerebrosidase activity, permeated the blood–brain barrier, and decreased glucosylsphingosine levels in the cerebrospinal fluid. Myoclonus, seizures, and pupillary light reflex dysfunction markedly improved in all participants. Relief from myoclonus led to impressive recovery of gross motor function in two participants, allowing them to walk again.
Aries 2022 [60]	β-Glucosidase	Binds in a mutation-dependent manner to misfolded proteins in the ER and facilitates the shuttle to the lysosome	Prospective; individual case study	Clinical and biochemical outcome of an individual with GD2 treated with high-dose ambroxol from the age of 4 months	Glucosylsphingosine (Lyso-GL1) in cerebrospinal fluid decreased remarkably compared to pre-treatment, whereas Lyso-GL1 and chitotriosidase in blood increased. Ambroxol treatment of participant fibroblasts revealed a significant increase in β-glucocerebrosidase activity in vitro. Combination of high-dose ambroxol with ERT proved to be a successful approach to manage both visceral and neurological manifestations.

Abbreviations: ERT, enzyme replacement therapy; GD, Gaucher disease; MoA, mode of action.

**Table 4:** Clinical evidence summary: Pompe disease.

Author Year (Reference)	Enzyme	MoA (as Per Author’s Comments)	Clinical Study Type	Study Objective	Summary Outcome
**NB-DNJ (miglustat) + alglucosidase alfa**					
Parenti 2014 [20]	Alglucosidase alfa	Miglustat in combination with exogenous recombinant alglucosidase alfa increases intracellular activity, facilitates lysosomal trafficking, maturation and stability of alglucosidase alfa in target cells.	Open, intra-patient, interventional study comparing miglustat + alglucosidase alfa vs. alglucosidase alfa (13 participants)	Effect on GAA activity	Combination treatment with miglustat + alglucosidase alfa resulted in enzyme activities greater than 1.85-fold the activities with alglucosidase alfa alone (2.19-fold increase at 12 h and 6.07-fold at 24 h and 3.95-fold at 36 h). Area under the curve was also significantly increased (6.78-fold *p* = 0.002). Results suggest improved stability of alglucosidase alfa in blood in the presence of the chaperone miglustat.
Miglustat + cipaglucosidase alfa					
Schoser 2021 [21]	Cipaglucosidase alfa	Miglustat in combination with exogenous cipaglucosidase alfa prevents the replacement enzyme from breaking down in the blood, so more of it is expected to get into the lysosomes improving the symptoms of the disease.	PROPEL (NCT03729362)—randomized controlled trial, double-blind, parallel group, phase III (125 participants)	To assess the safety and efficacy of an investigational two-component therapy (cipaglucosidase alfa, a novel recombinant human GAA, plus miglustat, an enzyme stabilizer) vs. alglucosidase alfa plus placebo for LOPD	Week 52, mean change from baseline in 6-minute walk distance was 20.8 m (SE 4.6) in the cipaglucosidase alfa plus miglustat group versus 7.2 m (6.6) in the alglucosidase alfa plus placebo group. Of the 123 participants, 118 (96%) experienced at least one treatment-emergent adverse event during the study; the incidence was similar between the cipaglucosidase alfa plus miglustat group (n = 81 (95%)) and the alglucosidase alfa plus placebo group (n = 37 (97%)). Cipaglucosidase alfa plus miglustat did not achieve statistical superiority to alglucosidase alfa plus placebo for improving 6-minute walk distance in the overall population of patients with LOPD.

Abbreviations: ERT, enzyme replacement therapy; FVC, forced vital capacity; LOPD, late-onset Pompe disease; MoA, mode of action; GAA, α-Glucosidase.

**Table 5:** Clinical evidence summary: GM2 gangliosidosis—Tay–Sachs disease.

Author Year (Reference)	Enzyme	MoA (as Per Author’s Comments)	Clinical Study Type	Study Objective	Summary Outcomes
**Pyrimethamine**					
Clarke 2011 [77]	Acid β-hexosaminidase	Depresses folate metabolism in participants receiving treatment with other folate inhibitors or agents associated with myelosuppression, including cotrimoxazole, trimethoprim, proguanil, zidovudine, or cytostatic agents (e.g., methotrexate).	Open-label phase I/II (11 participants)	Tolerability and efficacy with escalating doses of pyrimethamine	Pyrimethamine enhances leukocyte Hex A activity in people with late-onset GM2 gangliosidosis at doses lower than those associated with unacceptable side effects (e.g., 4-fold enhancement of Hex A activity at doses of 50 mg per day or less was observed).
Osher 2015 [78]	Acid β-hexosaminidase	Depresses folate metabolism in participants receiving treatment with other folate inhibitors or agents associated with myelosuppression, including cotrimoxazole, trimethoprim, proguanil, zidovudine, or cytostatic agents (e.g., methotrexate).	Open-label, extended pilot study (4 participants)	Tolerability and efficacy with cyclic, low-dose, long-term pyrimethamine	Hex A activity rose in all subjects, with a mean peak increase of 2.24-fold (SD ±0.52 over baseline activity, range 1.87–3). Mean treatment time required to attain this peak was 15.7 weeks (±4.8; SD). Following increased activity, Hex A gradually declined with the continued use of PMT. A second cycle of PMT treatment was then initiated, resulting again in an increase in Hex A activity. Three of the participants experienced a measurable neuropsychiatric deterioration, whereas one subject remained entirely stable.

Abbreviations: Hex A, α subunit of β-hexosaminidase enzyme; MoA, mode of action; PMT, pyrimethamine.

**Table 6:** Clinical evidence summary: NPC disease.

Author Year (Reference)	Enzyme	MoA (as Per Author’s Comments)	Clinical Study Type	Study Objective	Summary Outcomes
**Miglustat**					
Patterson 2007 [81]	Sphingomyelinase	Unclear	Randomized controlled trial (MIG vs. standard care) (29 participants aged 12 years and over and 12 participants aged under 12 years)	Efficacy and safety	At 12 months, HSEM velocity had improved in participants treated with miglustat versus those receiving standard care; results were significant when participants taking benzodiazepines were excluded (*p* = 0.028). Children showed an improvement in HSEM velocity of similar size at 12 months. Improvement in swallowing capacity, stable auditory acuity, and a slower deterioration in ambulatory index were also seen in treated participants older than 12 years. Miglustat 200 mg 3 times daily was well tolerated and consistent with that seen in trials in type 1 Gaucher disease (at half the dose).

Abbreviations: HSEM, horizontal saccadic eye movement; MIG, miglustat; MoA, mode of action.

**Table 7:** The small molecule chaperone (SMC) classification system—a new classification system to describe small molecule chaperones based on their action.

Classification	Chaperone	Stabilizer
**Use**	Monotherapy	Combined/coadministration
**Type of action**	Chaperone	Stabilizer
**Acts on**	Endogenous enzyme	Exogenously administered enzyme (ERT)
**Site of action** **(trafficking to/from)**	Intracellular (ER→Golgi→endosomes→lysosomes)	Circulation (bloodstream→cell uptake receptor→endosomes→lysosomes)
**Example**	Migalastat	Miglustat plus cipaglucosidase alfa

Abbreviations: ER, endoplasmic reticulum; ERT, enzyme replacement therapy.

## Data Availability

The data presented in this study are available in the Supplementary Materials described above.

## Supplementary Materials

The following supporting information can be downloaded at: https://www.mdpi.com/article/10.3390/biom13081227/s1. Figure S1: PRISMA figure; Table S1: Literature search strategy and study selection process; Tables S2–S7: Preclinical evidence summary: Fabry disease; Gaucher disease; Pompe disease; GM1 gangliosidosis—Morquio B disease; GM2 gangliosidosis—Tay–Sachs disease; Mucopolysaccharidosis I.
