# Supplementary material for: Therapeutic Role of Pharmacological Chaperones in Lysosomal Storage Disorders: A Review of the Evidence and Informed Approach to Reclassification
Source: Biomolecules. 2023 Aug 7;13(8):1227. doi: 10.3390/biom13081227 (PMC10452329; doi:10.3390/biom13081227)
Supplement: Supplementary file 1 [file biomolecules-13-01227-s001.zip › biomolecules-2489010-supplementary.pdf]

Figure S1. PRISMA flow diagram

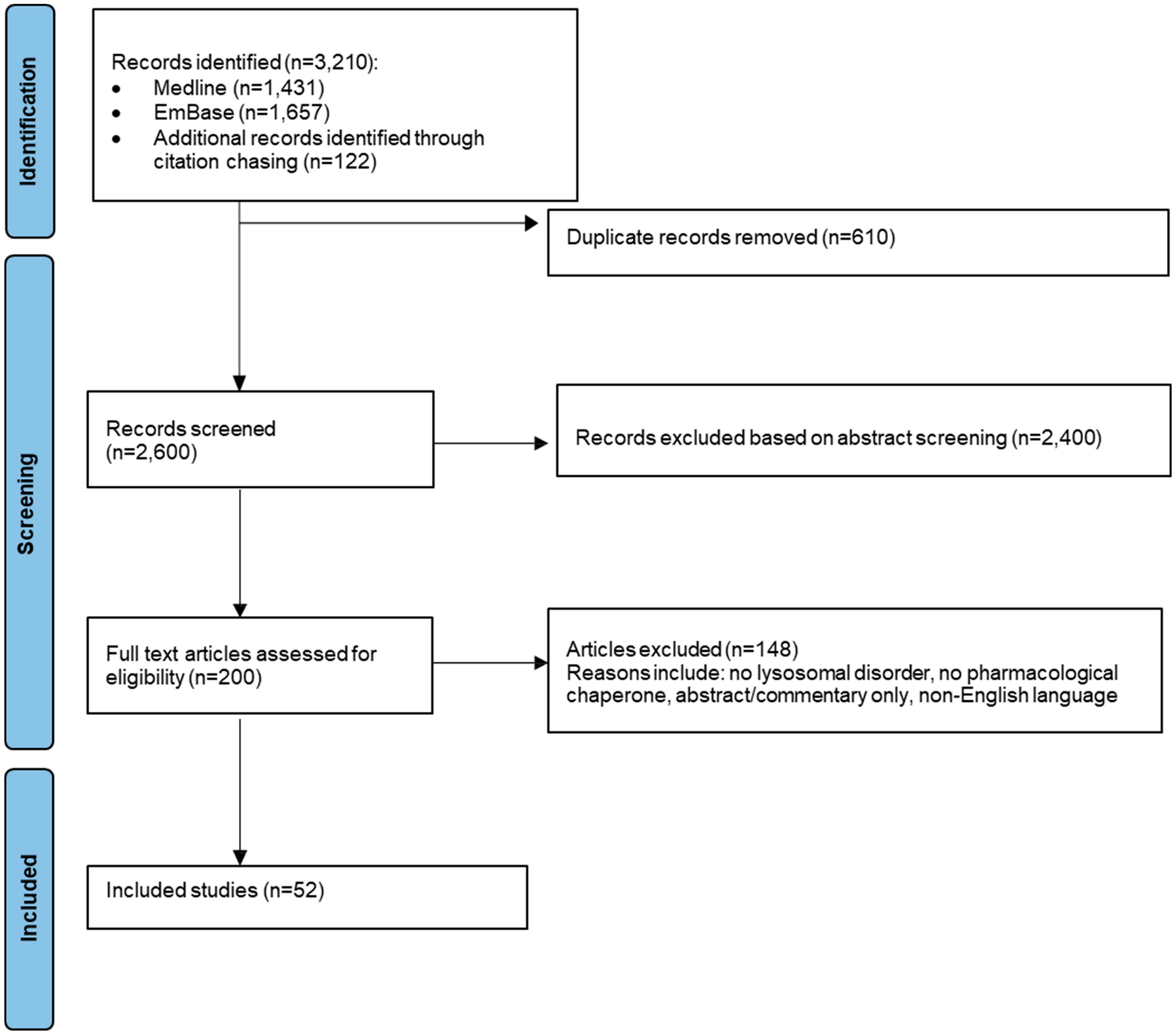

Table S1. Literature search strategy and study selection process

Field	Content
Objective	To conduct a systematic search to identify and map evidence on pharmacological chaperone therapies in lysosomal storage disorders
Searches	The following databases were searched:<br>• Embase<br>• MEDLINE All<br>No date or language limits were applied.
Condition or domain being studied	Chaperone therapies in lysosomal storage disorders
Population	People with lysosomal storage disorders
Interventions	• Chaperone therapies
Types of study to be included	• Any study design reporting information on a chaperone therapy in lysosomal storage disorders
Other exclusion criteria	Studies were not included for the following reasons:<br>Language:<br>• Non-English<br>Publication status:<br>• Abstract only<br>Study type:<br>• Commentary or non-systematic review article. (Note references included in commentary or review articles will not be assessed for relevance)<br>• Letters<br>• Editorials
Study selection	An initial sample of 10% of abstracts (n=253) was screened independently by two reviewers to pilot

Field	Content
	the inclusion criteria and ensure consistency prior to undertaking title and abstract screening. Inter-rater agreement was assessed and discrepancies were resolved by discussion. The remaining studies were single-screened. Study selection used the pre-defined inclusion/exclusion criteria outlined above. Citations identified as eligible during title/abstract review were retrieved in full-text for further review and screened in the same way. For the full-text screening phase, articles excluded were documented with reasons for their exclusion according to the pre-defined criteria (PICOS). Screening was conducted in Covidence.
Data reporting and synthesis	The evidence was categorized according to themes, e.g., studies reporting mechanism of action, application in lysosomal storage disorders.<br>Study characteristics were reported in tables with a descriptive summary of MoA, study type and objective, and summary outcomes (as no specific outcomes are specified in the PICO).
Key search terms	**Ovid MEDLINE(R) ALL <1946 to 2023 February 20>:**<br>1 molecular chaperones/ 19097<br>2 (chaperone$ or pharmacoperone$).ti,ab,ot. 37739<br>3 ((chaperone adj2 (therap* or pharmacolog* or molecular)) or pharmacoperone).ti,ab,kw. 6067<br>4 ((inhibitor* or non-inhibitor* or non inhibitor* or pharmacolog* or molecular*) adj2 chaperone*).ti,ab,kw. 10781<br>5 ((protein or enzym* or pharmacolog*) adj1 (stabilis* or stabiliz*)).ti,ab,kw. 3929<br>6 1 or 2 or 3 or 4 or 5 49821<br>7 "randomized controlled trial".pt. 555133<br>8 (random$ or placebo$ or single blind$ or double blind$ or triple blind$).ti,ab. 1381788<br>9 (retraction of publication or retracted publication).pt. 20971

Field	Content
	10 or/7-9 1504703
	11 (animals not humans).sh. 4905488
	12 ((comment or editorial or meta-analysis or practice-guideline or review or letter) not "randomized controlled trial").pt. 5014510
	13 (random sampl$ or random digit$ or random effect$ or random survey or random regression).ti,ab. not "randomized controlled trial".pt. 106987
	14 10 not (11 or 12 or 13) 1094733
	15 exp animals/ not humans.sh. 4939103
	16 14 not 151094637
	17 Epidemiologic studies/ 8955
	18 exp case control studies/ 1269638
	19 exp cohort studies/ 2275392
	20 Case control.tw. 139719
	21 (cohort adj (study or studies)).tw. 258202
	22 Cohort analy$.tw.9817
	23 (Follow up adj (study or studies)).tw. 52678
	24 (observational adj (study or studies)).tw. 133110
	25 longitudinal.tw. 282348
	26 Retrospective.tw. 634388
	27 Cross sectional.tw. 429207
	28 Cross-sectional studies/ 406127
	29 17 or 18 or 19 or 20 or 21 or 22 or 23 or 24 or 25 or 26 or 27 or 28 3420418
	30 review.pt. 2918450

Field	Content	
	31	(medline or medlars or embase or pubmed or cochrane).tw,sh. 279247
	32	(scisearch or psychinfo or psycinfo).tw,sh. 47330
	33	(psychlit or psyclit).tw,sh. 917
	34	cinahl.tw,sh. 35567
	35	((hand adj2 search$) or (manual$ adj2 search$)).tw,sh. 14990
	36	(electronic database$ or bibliographic database$ or computeri?ed database$ or online database$).tw,sh. 47644
	37	(pooling or pooled or mantel haenszel).tw,sh. 126378
	38	(peto or dersimonian or der simonian or fixed effect).tw,sh. 9029
	39	(retraction of publication or retracted publication).pt. 20971
	40	31 or 32 or 33 or 34 or 35 or 36 or 37 or 38 or 39 432455
	41	30 and 40 183404
	42	meta-analysis.pt. 150108
	43	meta-analysis.sh. 150108
	44	(meta-analys$ or meta analys$ or metaanalys$).tw,sh. 251976
	45	(systematic$ adj5 review$).tw,sh. 261928
	46	(systematic$ adj5 overview$).tw,sh. 2887
	47	(quantitativ$ adj5 review$).tw,sh. 9327
	48	(quantitativ$ adj5 overview$).tw,sh.370
	49	(quantitativ$ adj5 synthesis$).tw,sh.3723
	50	(methodologic$ adj5 review$).tw,sh. 7445
	51	(methodologic$ adj5 overview$).tw,sh. 490
	52	(integrative research review$ or research integration).tw. 156

Field	Content
	53 42 or 43 or 44 or 45 or 46 or 47 or 48 or 49 or 50 or 51 or 52 401934
	54 41 or 53 473231
	55 economics/ 27408
	56 exp "costs and cost analysis"/ 253013
	57 economics, nursing/ 4010
	58 economics, medical/ 9173
	59 economics, pharmaceutical/ 3048
	60 exp economics, hospital/ 25444
	61 economics, dental/ 1920
	62 exp "fees and charges"/ 31011
	63 exp budgets/ 13951
	64 budget*.ti,ab,kf. 32801
	65 (economic* or cost or costs or costly or costing or price or prices or pricing or pharmacoeconomic* or pharmaco-economic* or expenditure or expenditures or expense or expenses or financial or finance or finances or financed).ti,kf. 254014
	66 (economic* or cost or costs or costly or costing or price or prices or pricing or pharmacoeconomic* or pharmaco-economic* or expenditure or expenditures or expense or expenses or financial or finance or finances or financed).ab. /freq=2 333412
	67 (cost* adj2 (effective* or utilit* or benefit* or minimi* or analy* or outcome or outcomes)).ab,kf. 184931
	68 (value adj2 (money or monetary)).ti,ab,kf. 2718
	69 exp models, economic/ 15989
	70 economic model*.ab,kf. 3750

Field	Content
	71 markov chains/ 15516
	72 markov.ti,ab,kf. 25659
	73 monte carlo method/ 30695
	74 exp Decision Theory/ 12754
	75 monte carlo.ti,ab,kf. 54490
	76 (decision* adj2 (tree* or analy* or model*)).ti,ab,kf. 29809
	77 or/55-76 809842
	78 16 or 29 or 54 or 77 5247472
	79 6 and 78 1431
	**Embase <1974 to 2022 January 06>:**
	1 molecular chaperones/ 30402
	2 (chaperone$ or pharmacoperone$).ti,ab,ot. 45768
	3 ((chaperone adj2 (therap* or pharmacolog* or molecular)) or pharmacoperone).ti,ab,kw. 7538
	4 ((inhibitor* or non-inhibitor* or non inhibitor* or pharmacolog* or molecular*) adj2 chaperone*).ti,ab,kw. 12983
	5 ((protein or enzym* or pharmacolog*) adj1 (stabilis* or stabiliz*)).ti,ab,kw. 4679
	6 1 or 2 or 3 or 4 or 5 56031
	7 Economics/ 243091
	8 cost/ 60227
	9 exp Health Economics/ 938120
	10 Budget/ 31133
	11 budget*.ti,ab,kw. 42676
	12 (economic* or cost or costs or costly or costing or price or prices or pricing or

Field	Content
	pharmacoeconomic* or pharmaco-economic* or expenditure or expenditures or expense or expenses or financial or finance or finances or financed).ti,kw. 282497<br>13 (economic* or cost or costs or costly or costing or price or prices or pricing or pharmacoeconomic* or pharmaco-economic* or expenditure or expenditures or expense or expenses or financial or finance or finances or financed).ab./freq=2 9861<br>14 (cost* adj2 (effective* or utilit* or benefit* or minimi* or analy* or outcome or outcomes)).ab,kw. 251639<br>15 (value adj2 (money or monetary)).ti,ab,kw. 3645<br>16 Statistical Model/ 168444<br>17 economic model*.ab,kw. 5449<br>18 Probability/ 124567<br>19 markov.ti,ab,kw. 31950<br>20 monte carlo method/ 44941<br>21 monte carlo.ti,ab,kw. 54560<br>22 Decision Theory/ 1797<br>23 Decision Tree/ 16374<br>24 (decision* adj2 (tree* or analy* or model*)).ti,ab,kw. 40624<br>25 or/7-24 1662359<br>26 (random$ or placebo$ or single blind$ or double blind$ or triple blind$).ti,ab. 1877272<br>27 RETRACTED ARTICLE/ 11093<br>28 26 or 27 1887890<br>29 (animal$ not human$).sh,hw. 4576813<br>30 (book or conference paper or editorial or letter or review).pt. not exp randomized controlled trial/ 5469754<br>31 (random sampl$ or random digit$ or random effect$ or random survey or random regression).ti,ab. not exp randomized controlled trial/ 135795

Field	Content
	32 28 not (29 or 30 or 31) 1440436<br>33 Clinical study/ 156942<br>34 case control study/ 181990<br>35 Family study/ 25364<br>36 Longitudinal study/ 165450<br>37 Retrospective study/ 1181108<br>38 Prospective study/ 736268<br>39 Randomized controlled trials/ 217550<br>40 38 not 39727762<br>41 Cohort analysis/ 791764<br>42 (Cohort adj (study or studies)).mp. 380843<br>43 (Case control adj (study or studies)).tw. 150091<br>44 (follow up adj (study or studies)).tw. 67991<br>45 (observational adj (study or studies)).tw. 206379<br>46 (epidemiologic$ adj (study or studies)).tw. 113949<br>47 (cross sectional adj (study or studies)).tw. 273079<br>48 33 or 34 or 35 or 36 or 37 or 40 or 41 or 42 or 43 or 44 or 45 or 46 or 47 3275867<br>49 exp review/ 2868061<br>50 (literature adj3 review$).ti,ab. 396997<br>51 exp meta analysis/ 234529<br>52 exp 'Systematic Review'/ 326957<br>53 49 or 50 or 51 or 52 3185477<br>54 (medline or medlars or embase or pubmed or cinahl or amed or psychlit or psyclit or psychinfo or psycinfo or scisearch or cochrane).ti,ab. 363248<br>55 RETRACTED ARTICLE/ 11093<br>56 54 or 55 373966

Field	Content
	57 53 and 56 288779<br>58 (systematic$ adj2 (review$ or overview)).ti,ab. 302359<br>59 (meta?anal$ or meta anal$ or meta-anal$ or metaanal$ or metanal$).ti,ab. 286066<br>60 57 or 58 or 59 543955<br>61 25 or 32 or 48 or 60 6193221<br>62 6 and 61 1657

Table S2. Preclinical evidence summary: Fabry disease

The chaperone galactose for α-Galactosidase A (α-Gal A) increased the cellular activity of different missense mutant forms of α-Gal A in vitro with concomitant reductions in glycosphingolipid globotriaosylceramide (Gb3/GL-3) (Okuyima 1995 (1)). The chaperone 1-deoxygalactonojirimycin (DGJ, AT1001, migalastat) for α-Gal A increased cellular activity of different missense mutant forms of α-Gal A in vitro and/or ex vivo (Andreotti 2011 (2); Asano 2000 (3); Fan 1999 (4); Lukas 2020 (5); Sugawara 2008 (6); Wu 2011 (7)), with concomitant reductions in GL-3 and reduced globotriaosylsphingosine (lyso-Gb3) accumulation (Benjamin 2009 (8); Lukas 2013 (9); Porto 2012 (10); Seemann 2020 (11); Yu 2014 (12)). In addition, enhanced α-Gal A was observed in people with Fabry and R301Q and Q279E mutations (Fan 1999 (4)). Furthermore, DGJ acted synergistically with the proteostasis regulator 4-phenyl-butyrate (4-PBA) in in vitro and ex vivo Fabry disease models (Yu 2014 (12)).

Author Year (Reference)	Enzyme	MoA (as per author's comments)	Preclinical study type	Study objective	Summary outcome
**Galactose**					
Okuyima 1995 **(1)**	α-Gal A	Stabilisation against accelerated intracellular degradation (protection from intracellular inactivation and degradation).	Ex vivo models	Effect on α-Gal A activity	Many missense mutations in the α-Gal A gene allow the expression of catalytically active mutant enzymes, regardless of clinical phenotype, which are rapidly degraded under physiological conditions and stabilised by galactose.
**1-deoxygalactonojirimycin (DGJ); migalastat**					
Andreotti 2011 **(2)**	α-Gal A	The therapeutical approach with pharmaceutical chaperones relies on competitive inhibitors of α-Gal A that bind and stabilise the enzyme, increasing its total cellular levels, as shown in cultured cells and in vivo. Mutant lysosomal α-Gal A molecular subtypes were	Ex vivo models	Effect on α-Gal A activity	Transient transfection with 1-DGJ led to a 2.1- to 8.9-fold increase in α-Gal A expression, for the 9 molecular subtypes tested in vitro. This study provides experimental support to an 'in silico' method designed to predict missense mutations in the gene encoding lysosomal α-Gal A responsive to pharmacological chaperones.

Author Year (Reference)	Enzyme	MoA (as per author's comments)	Preclinical study type	Study objective	Summary outcome
		produced by site-directed mutagenesis and expressed in mammalian cells. The enzymatic activity of these mutants after 1-DGJ administration was assessed.			
Asano 2000 **(3)**	α-Gal A	DGJ is a potent inhibitor of α-Gal A at subinhibitory intracellular concentrations. It serves as a specific pharmacological chaperone for those mutant enzymes that failed to maintain their proper conformation to avoid excessive degradation.	Ex vivo models	Effect on α-Gal A activity	Inclusion of 1-DGJ at 100 μm in culture medium of Fabry R301Q lymphoblasts increased the intracellular α-Gal A activity by 14-fold. The establishment of the correlation between in vitro inhibition and intracellular enhancement provided a molecular basis for a general method to screen specific chemical chaperones for other genetic disorders resulting from folding defection of proteins.
Benjamin 2009 **(8)**	α-Gal A	DGJ is a pharmacological chaperone that selectively binds α-Gal A, increasing physical stability, lysosomal trafficking, and cellular activity.	Ex vivo models	Effect on α-Gal A activity	In cultured fibroblasts from males with Fabry disease, the responses to DGJ were comparable to those of lymphoblasts with the same mutation.<br>Increases in α-Gal A levels (1.5- to 28-fold) after continuous DGJ incubation for 5 days were seen for 49 different missense mutant forms.<br>Elevated GL-3 levels in responsive Fabry fibroblasts were reduced after DGJ incubation, indicating that increased mutant α-Gal A levels can reduce accumulated substrate.
Fan 1999 **(4)**	α-Gal A	DGJ is a potent competitive inhibitor of α-Gal A. It acts as a 'chaperone' to force the mutant enzyme to assume the proper conformation. It might specifically protect the	In vitro and ex vivo models	Effect on α-Gal A activity	DGJ enhanced mutant α-Gal A activities in lymphoblasts from Fabry patients with R301Q or Q279E mutations (up to 8-fold and 7-fold, respectively). These results indicate administration of α-Gal A inhibitor may be used as a new molecular therapy for Fabry disease with

Author Year (Reference)	Enzyme	MoA (as per author's comments)	Preclinical study type	Study objective	Summary outcome
		catalytic center of α-Gal A and reduce the flexibility of folding in situ, thus leading the mutant α-Gal A to a proper conformation, whose transport would not be retarded.			mutations at sites other than the catalytic center.
Lukas 2013 **(9)**	α-Gal A	α-Gal A activity was measured and responsiveness to DGJ was tested to determine the likelihood of newly identified GLA variants leading to FD.	Ex vivo models	Effect on α-Gal A activity	For patients with GLA mutations where there was still >6% residual α-Gal A activity, DGJ led to elevated mutant α-Gal A activity and reduced lyso-Gb3 accumulation. By analyzing a significant number of mutations and correlating these with the clinical manifestation related to the mutation, this enables us to provide information for the clinical relevance of PC therapy for a given mutant.
Lukas 2020 **(5)**	α-Gal A	Oral DGJ treatment increases α-Gal A activity in patients carrying biochemically responding, amenable gene variants in the encoding gene (GLA).	Ex vivo models	Effect on α-Gal A activity	A comparison of two classification methods of amenability to DGJ treatment showed some inter-assay discrepancy, but was generally highly reproducible. Particular care should be taken in ascertaining a treatment decision in cases of low baseline activity, borderline biochemical responsiveness.
Porto 2012 **(10)**	α-Gal A	Co-administration of enzyme replacement therapy (ERT) and PC to substantially increase in the amount of intracellular enzyme compared with cells treated with ERT alone. This suggests improved intracellular	Ex vivo models (fibroblasts)	Effect of ERT+DGJ on α-Gal A activity	This study provides in vitro evidence that co-administration of ERT and PC resulted in a better correction (4.8 to 16.9-fold) of intracellular α-Gal A activity. Co-administration protocols may be superior to ERT alone. Although these studies require further confirmation in vivo, the results hold promise for the treatment of FD patients and suggest that this approach can be extended to any

Author Year (Reference)	Enzyme	MoA (as per author's comments)	Preclinical study type	Study objective	Summary outcome
		stability of the enzyme.			other LSD for which ERT and chaperones are available.
Seemann 2020 **(11)**	α-Gal A	The identification of candidate small proteostasis regulator molecules able to elevate mutant α-Gal A activity and reduce lyso-Gb3 in patient-derived fibroblasts and understanding of the mechanisms involved.	In vitro and ex vivo models	Characterisation of chaperones	Addressing proteostasis is an effective approach to discovering new therapeutic targets for diseases involving folding and trafficking-deficient protein mutants.
Sugawara 2008 **(6)**	α-Gal A	Examination of structural changes in GLAs due to amino acid substitutions by determining the number of atoms affected, the root-mean-square distance (RMSD), and the solvent-accessible surface area (ASA). Structural changes due to amino acid substitutions for which substrate analogues are effective tend to be small and located on the surface of the GLA molecule.	In vitro and ex vivo models	Identification of mutant forms of α-Gal A activity	Structural investigation is useful for elucidation of the basis of Fabry disease, and it will increase our ability to determine a proper therapeutic schedule for this disease.
Wu 2011 **(7)**	α-Gal A	Migalastat selectively binds and stabilises α-Gal A, increasing total cellular levels and activity for some missense mutant forms of α-Gal A (those responsive to the PC were determined by a cell-	In vitro and ex vivo models	Identification of mutant forms of α-Gal A activity	This assay identifies mutant forms of α-Gal A that respond to migalastat (AT1001) in patient-derived cell lines and in PBMCs of Fabry patients administered the drug. Thus, it may prove to be a useful complementary aid in the identification of Fabry patients with AT1001-responsive mutant forms.

Author Year (Reference)	Enzyme	MoA (as per author's comments)	Preclinical study type	Study objective	Summary outcome
		based assay).			
Yu 2014 **(12)**	α-Gal A	Stabilisation of mutant α-Gal A against premature degradation and restoration of activity and trafficking; reduced Gb3 substrate accumulation and autophagy impairments in FD cell cultures.	In vitro and ex vivo models	Effect on α-Gal A activity	In vitro results for this new family of PCs show high efficacy in enhancing the residual activity of FD-associated α-Gal A mutants, reducing the accumulation of the substrate Gb3 in FD cells. Moreover, they acted synergistically with the proteostasis regulator 4-PBA, appearing to be promising leads as pharmacological chaperones for FD.

Abbreviations: α-Gal A, α-Galactosidase A; DGJ, chaperone 1-deoxygalactonojirimycin; Gb3/GL3, glycosphingolipid globotriaosylceramide; GLA, α-galactosidase A; lyso-Gb3; globotriaosylsphingosine; ERT, enzyme replacement therapy; FD, Fabry disease; LSD, lysosomal storage disorder; PBMCs, peripheral blood mononuclear cells; PC, pharmacological chaperone; 4-PBA, 4-phenyl-butyrate.

Table S3. Preclinical evidence summary: Gaucher disease

In Gaucher disease, isofagomine (IFG) is an azasugar that binds both wild-type and mutant forms of GCase, resulting in stabilization and increased cellular and lysosomal levels (Dasgupta 2015 (13); Khanna 2010 (14); Sun 2012 (15)). Overall, IFG administration (at different doses) led to statistically significant increases in glucocerebrosidase (Gcase) activity in liver, spleen, lung, bone, and brain, as well as in liver macrophages (Dasgupta 2015 (13); Khanna 2010 (14); Sun 2012 (15)). In the L444P model, IFG administration resulted in a decrease in plasma chitin III and immunoglobulin G (IgG) levels (Khanna 2010 (14)) and reduced liver and spleen weights, providing evidence of in vivo efficacy (Khanna 2010 (14)). Preclinical in vivo studies evaluated the effects of isofagomine in mouse models of Gaucher disease with mutation L444P (Khanna 2010 (14)). The chaperone 1-deoxynojirimycin (DNJ) promoted GCase activity in Gaucher fibroblasts hosting the neuronopathic G188S/G183W mutation (Mena-Barrgan 2018 (16)). The chaperone arimoclomol was shown to enhance the folding, maturation, activity, and correct cellular localisation of mutated GCase across several genotypes including the common L444P and N370S mutations in primary cells from Gaucher disease patients (Fog 2018 (17)). Fibroblasts harbouring the G202R and/or L444P mutations were stable at 37 °C in the presence of the chaperone NN-DNJ (Sawkar 2002 (18); Sawkar 2005 (19); Sawkar 2006 (20)). The chaperone ambroxol showed pH-dependent affinity for GCase, with decreasing inhibition at lysosomal pH values. In in vitro studies, ambroxol elevated the cellular and lysosomal levels of multiple GCase mutants in patient-derived cells (Bednikov-Bar 2013 (21)), including those expressing F213I or N370S (Maegawa 2009 (22)). The chaperone NCGC607 decreased glucocerebrosidase activity and stored the glycolipid substrates glucosylceramide and glucosylsphingosine (Aflaki 2016 (23)).

Author Year (Reference)	Enzyme	MoA (as per author's comments)	Study type	Study objective	Summary outcomes
**Isofagomine**					
Dasgupta 2015 **(13)**	β-Glucosidase	An iminosugar, binds selectively to N370S glucocerebrosidase and restores its correct	Mouse models	Effect on substrate accumulation, sensorimotor function, neuron and microglial cell status, and	IFG treatment did not alter the glucosylsphingosine (GS) and glucosylceramide (GC) accumulation significantly but attenuated the progression of the disease and altered numerous differentially expressed

Author Year (Reference)	Enzyme	MoA (as per author's comments)	Study type	Study objective	Summary outcomes
		conformation and enhances its activity		mitochondrial function. Change in brain mRNA and miRNA	brain miRNAs (DEmiRs) and target differentially expressed brain mRNAs (DEGs) to their respective normal levels in inflammation, mitochondrial function, and axonal guidance pathways, suggesting its regulation of miRNA and the associated mRNA that underlie the neurodegeneration in neuronopathic Gaucher disease (nGD).
Khanna 2010 **(14)**	β-Glucosidase	An iminosugar, binds selectively to N370S glucocerebrosidase and restores its correct conformation and enhances its activity	Mouse models	Effect on L444P GCase activity and GCase levels	In mice expressing murine L444P GCase, oral administration of IFG resulted in significant increases (2- to 5-fold) in GCase activity in disease-relevant tissues, including brain. Additionally, eight-week IFG administration significantly lowered plasma chitin III and IgG levels, and 24-week administration significantly reduced spleen and liver weights. Taken together, these data suggest that IFG can increase the lysosomal activity of L444P GCase in cells and tissues.
Sun 2012 **(15)**	β-Glucosidase	An iminosugar, binds selectively to N370S glucocerebrosidase and restores its correct	GCase mutant fibroblasts and mouse models	Effect on GCase activity and impact on substrate levels	The liver-secreted human GCase in serum was stabilised, and its effect on the lung and spleen involvement was enhanced by IFG treatment. In 8-week IFG-treated mice, the accumulated glucosylceramide and

Author Year (Reference)	Enzyme	MoA (as per author's comments)	Study type	Study objective	Summary outcomes
		conformation and enhances its activity			glucosylsphingosine were reduced by 75 and 33%, respectively. Decreases of storage cells were correlated with >50% reductions in substrate levels. These results indicate that IFG stabilises GCase in tissues and serum and can reduce visceral substrates in vivo.
**1-deoxynojirimycin (DNJ)**					
Mena Barragan 2018 **(16)**	β-Glucosidase	Suppression of intestinal α-1,4-glucosidase as well as α-1,6-glucosidase of hepatic glycogen-debranching enzymes leading to a reduced rate of oligosaccharide breakdown	Ex vivo models	Effect on GCase activity and impact on substrate levels	The 1-deoxynojirimycin (DNJ)-related nonreducing conjugates behaved as stronger GCase inhibitors than the reducing counterparts and exhibited potent chaperoning capabilities in Gaucher fibroblasts hosting the neuronopathic G188S/G183W mutation, the isothiourea derivative being indeed one of the most efficient chaperone candidates reported to date (70% activity enhancement at 20 pM). At their optimal concentration, the four selected compounds promoted mutant GCase activity enhancements over 3-fold; yet, the inhibitor/chaperoning balance became unfavorable at much lower concentration for nonreducing as compared to reducing derivatives.
**Arimoclomol**					
Fog 2018 **(17)**	β-Glucosidase	Arimoclomol is	Ex vivo models (skin	Effect on GCase	Arimoclomol induced relevant heat

Author Year (Reference)	Enzyme	MoA (as per author's comments)	Study type	Study objective	Summary outcomes
		believed to function by stimulating a normal cellular protein repair pathway through the activation of molecular chaperones	fibroblasts derived from patients)	activity in ex vivo systems of primary fibroblasts and neuronal-like cells	shock proteins (HSPs) such as ER-resident HSP70 (BiP) and enhanced the folding, maturation, activity, and correct cellular localisation of mutated GCase across several genotypes including the common L444P and N370S mutations in primary cells from GD patients. These effects where recapitulated in a human neuronal model of GD obtained by differentiation of multipotent adult stem cells.
**NN-DNJ**					
Sawkar 2002 **(18)**	β-Glucosidase	Unclear	Ex vivo models	Impact on WT β-glucosidase activity	NN-DNJ chaperone increases WT β-glucosidase activity, but not that of L444P, a less prevalent Gaucher disease variant. Incubation of isolated soluble WT enzyme with NN-DNJ reveals that β-glucosidase is stabilised against heat denaturation in a dose-dependent fashion.
Sawkar 2006 **(20)**	β-Glucosidase	Unclear	Ex vivo models	Impact of changes, e.g., temperature in the endoplasmic reticulum environment	N370S, L444P, and G202R GC are destabilised in the neutral pH environment of the ER, rendering them prone to ERAD. Fibroblasts harboring the G202R and L444P GC mutations grown at 30 °C localise the mutant proteins to the lysosome, and this increases total GC activity. Both of these temperature-sensitive

Author Year (Reference)	Enzyme	MoA (as per author's comments)	Study type	Study objective	Summary outcomes
					mutants appear to be stable at 37 °C once they are trafficked to the low pH environment of the lysosome.
Sawkar 2005 **(19)**	β-Glucosidase	Unclear	Ex vivo models	Impact on glucocerebrosidase variants	NN-DNJ chaperone also increases WT β-glucosidase activity, but not that of L444P, a less prevalent Gaucher disease variant. Incubation of isolated soluble WT enzyme with NN-DNJ reveals that β-glucosidase is stabilised against heat denaturation in a dose-dependent fashion.
**Ambroxol**					
Bendikov-Bar 2013 **(21)**	β-Glucosidase	Ambroxol can elevate the lysosomal fraction and enzymatic activity of several mutant GCase variants	Ex vivo models (skin fibroblasts derived from patients)	Effect on lysosomal fraction and enzymatic activity of mutant GCase variants	Ambroxol increases both the lysosomal fraction and the enzymatic activity of several mutant GCase variants in skin fibroblasts derived from type 1 and type 2 GD patients.
Maegawa 2009 **(22)**	β-Glucosidase	Potent stabiliser of GCase and a pH-dependent, mixed inhibitor of the enzyme	Animal models	Identification and characterisation of ambroxol	Treatment significantly increased N370S and F213I mutant GCase activity and protein levels in GD fibroblasts. These increases were primarily confined to the lysosome-enriched fraction of treated cells, a finding confirmed by confocal immunofluorescence microscopy. Additionally, enhancement of GCase activity and a reduction in glucosylceramide storage was verified

Author Year (Reference)	Enzyme	MoA (as per author's comments)	Study type	Study objective	Summary outcomes
					in ABX-treated GD lymphoblasts (N370S/N370S). Hydrogen/deuterium exchange mass spectrometry revealed that upon binding of ambroxol, amino acid segments 243–249, 310–312, and 386– 400 near the active site of GCase are stabilised. Consistent with its mixed type inhibition of GCase, modeling studies indicated that ambroxol interacts with both active and non-active site residues.
**NCGC607**					
Aflaki 2016 **(23)**	β-Glucosidase	Unclear	Ex vivo models	Effect on glucocerebrosidase activity and protein levels, and reduced glycolipid storage	Cells exhibited decreased glucocerebrosidase activity and stored the glycolipid substrates glucosylceramide and glucosylsphingosine, demonstrating their similarity to patients with Gaucher disease. Dopaminergic neurons from patients with type 2 and type 1 Gaucher disease with parkinsonism had reduced dopamine storage and dopamine transporter reuptake. Levels of -synuclein, a protein present as aggregates in Parkinson's disease and related synucleinopathies, were selectively elevated in neurons from the patients with parkinsonism or type 2 Gaucher disease. The cells were then treated

Author Year (Reference)	Enzyme	MoA (as per author's comments)	Study type	Study objective	Summary outcomes
					with NCGC607, a small-molecule non-inhibitory chaperone of glucocerebrosidase identified by high-throughput screening and medicinal chemistry structure optimisation.

Abbreviations: DNJ, 1-deoxynojirimycin; GCase, glucocerebrosidase; GD, Gaucher disease; IFG, isofagomine; NN-DNJ, N-Nonyldeoxynojirimycin; miRNA, micro RNA; RNA, ribonucleic acid.

Table S4. Preclinical evidence summary: Pompe disease

Small-molecule chaperones have been proposed as a potential alternative to enzyme replacement therapy (ERT) for the treatment of Pompe disease. The chaperone N-butyldeoxynojirimycin (NB-DNJ, miglustat) stabilises recombinant human acid α-glucosidase (GAA), increases endogenous GAA activity in Pompe patient-derived fibroblasts, and increases activity of transiently expressed wild-type and mutant GAA in mammalian cells (Flangan 2009 (24); Okuyima 2007 (25); Porto 2009 (26); Parenti 2007 (27)). Furthermore, coincubation of NB-DNJ and recombinant human GAA increased α-glucosidase delivery to lysosomes, enhanced enzyme maturation, and increased enzyme stability (Porto 2009 (26)).

Author Year (Reference)	Enzyme	Mechanism of action	Study type	Study objective	Summary outcome
**NB-DNJ (miglustat)**					
Okumiya 2007 **(25)**	Acid α-Glucosidase	Acts as a chemical chaperone to restore the transport, maturation and activity of lysosomal acid α-glucosidase in a dose dependent manner, while it had no effect on the reference enzyme β-hexosaminidase	Ex vivo models (skin fibroblasts derived from patients)	Effect on acid α-glucosidase activity	NB-DNJ acts in low concentration as a pharmacological chaperone for certain mutant forms of acid α-glucosidase that are trapped in the endoplasmic reticulum, poorly transported, or labile in the lysosomal environment.
Porto 2009 **(26)**	Acid α-Glucosidase	Improves alpha-glucosidase delivery to lysosomes, enhances enzyme maturation, and increases enzyme stability; NB-DNJ effects may be variably influenced by genotype, disease severity, and underlying abnormalities of vesicles and membrane protein trafficking	Ex vivo models	Effect on acid α-glucosidase activity	Improved α-glucosidase delivery to lysosomes, enhanced enzyme maturation, and increased enzyme stability. Improved enzyme correction was also found in vivo in a mouse model of PD treated with co-administration of single infusions of recombinant

Author Year (Reference)	Enzyme	Mechanism of action	Study type	Study objective	Summary outcome
					human α-glucosidase and oral NB-DNJ.
**1-deoxynojirimycin (DNJ)**					
Flanagan 2009 **(24)**	Acid α-Glucosidase	An inhibitor of the ceramide-specific glycosyltransferase, which catalyzes the first step of glycosphingolipid biosynthesis and is currently approved for the oral treatment of type 1 GD	Ex vivo models	Effect on acid α-glucosidase activity	DNJ significantly increased enzyme activity and protein levels for 16 different GAA mutants in patient-derived fibroblasts and in transiently transfected COS-7 cells. Additionally, DNJ increased the processing of these acid-α-glucosidase (GAA) mutants to their mature lysosomal forms, suggesting it facilitated trafficking through the secretory pathway. Immunofluorescence microscopy studies showed increased colocalization of GAA with the lysosomal marker LAMP2 after incubation with DNJ, confirming increased lysosomal trafficking. A GAA structural model was constructed based on the related eukaryotic glucosidase maltase-glucoamylase. The mutated residues identified in

Author Year (Reference)	Enzyme	Mechanism of action	Study type	Study objective	Summary outcome
					responsive forms of GAA are located throughout most of the structural domains, with half of these residues located in two short regions within the catalytic domain.
**1-deoxynojirimycin (DNJ) and NB-DNJ (miglustat)**					
Parenti 2007 **(27)**	α-Glucosidase	An inhibitor of the ceramide-specific glycosyltransferase, which catalyzes the first step of glycosphingolipid biosynthesis and is currently approved for the oral treatment of type 1 GD	Ex vivo models (skin fibroblasts derived from patients)	Effect on acid α-glucosidase activity	Significant increase in GAA activity (1.3–7.5-fold) after iminosugar treatment in fibroblasts from patients carrying the mutations L552P and G459R. GAA enhancement was confirmed in HEK293T cells where the same mutations were overexpressed. No increase in GAA activity was observed for the other mutations.

Abbreviations: DNJ, 1-deoxynojirimycin; NB-DNJ, N-butyldeoxynojirimycin/miglustat; GAA, acid α-Glucosidase; COS, cells derived from CV-1; GD, Gaucher disease;

Table S5. Preclinical evidence summary: GM1-gangliosidosis – Morquio B disease

In GM1 gangliosidosis, the small molecule N-octyl-4-epi-β-valienamine (NOEV) was shown to significantly enhance β-galactosidase activity in mouse models of the disease (Matsuda 2003 (28); Suzuki 2007 (29)).

Reference	Enzyme	MoA (as per author's comments)	Study type	Study objective	Summary outcomes
**N-octyl-4-epi-β-valienamine (NOEV)**					
Matsuda 2003 **(28)**	Acid β-galactosidase	Unclear	Mouse model	Effect on beta-galactosidase activity	In R201C gangliosidosis, enhancement of the enzyme activity in the brain and other tissues. Decrease in the amount of GM1 and GA1 in neuronal cells in the fronto-temporal cerebral cortex and brainstem was observed.
Suzuki 2007 **(29)**	Acid β-galactosidase	Unclear	Mouse model	Effect on beta-galactosidase activity	Delivered rapidly to the brain, increased β-galactosidase activity, decreased ganglioside GM1, and prevented neurological deterioration within a few months. No adverse effect was observed.

Abbreviations: GM1, gangliosidosis, beta-galactosidosis; GA1, a derivative of GM1.

Table S6. Preclinical evidence summary: GM2 gangliosidosis – Tay–Sachs disease

The chaperone pyrimethamine was shown to significantly enhance β-galactosidase activity in fibroblast cells of patients with GM2 gangliosidosis (Tay–Sachs disease) (Maegawa 2007 (30)).

Author Year (Reference)	Enzyme	MoA (as per author's comments)	Study type	Study objective	Summary outcomes
**Pyrimethamine**					
Maegawa 2007 **(30)**	Acid β-hexosaminidase	Depresses folate metabolism in patients receiving treatment with other folate inhibitors, or agents associated with myelosuppression, including cotrimoxazole, trimethoprim, proguanil, zidovudine, or cytostatic agents (e.g., methotrexate).	Ex vivo models (cell lines derived from patients)	Effect on residual Hex A activity	Cells responding to PC treatment included those carrying mutants resulting in reduced Hex heat stability and partial splice junction mutations of the inherently less stable α-subunit. PYR, which binds to the active site in domain II, was able to function as PC even to domain I β-mutants. We concluded that PYR functions as a mutation-specific PC, variably enhancing residual lysosomal Hex A levels in late-onset GM2 gangliosidosis patient cells.

Abbreviations: Hex A, α subunit of β-hexosaminidase enzyme; PC, pharmacological chaperone; PYR, pyrimethamine.

Table S7. Preclinical evidence summary: Mucopolysaccharidosis I

In mucopolysaccharidosis I (MPS I), the chaperone iduronyl triazole-based analogs were shown to stabilise exogenous rh-α-IDUA protein in vitro (Cheng 2018 (31)).

Author Year (Reference)	Enzyme	MoA (as per author's comments)	Study type	Study objective	Summary outcomes
**Iduronyl triazole-based analogues**					
Cheng 2018 **(31)**	α-L-iduronidase	Unclear	In vitro	Identification and characterisation of chaperone	Small molecules can be used to stabilise exogenous rh-α-IDUA protein in vitro.

Abbreviation: rh-α-IDUA, recombinant humanised, alpha-L-iduronidase.

References

1. Okumiya T, Ishii S, Takenaka T, Kase R, Kamei S, Sakuraba H, Suzuki Y. Galactose stabilizes various missense mutants of alpha-galactosidase in Fabry disease. Biochem Biophys Res Commun. 1995;214(3):1219-24. 10.1006/bbrc.1995.2416.
2. Andreotti G, Citro V, De Crescenzo A, Orlando P, Cammisa M, Correra A, Cubellis MV. Therapy of Fabry disease with pharmacological chaperones: from in silico predictions to in vitro tests. Orphanet J Rare Dis. 2011;6:66. 10.1186/1750-1172-6-66.
3. Asano N., Ishii S., Kizu H., et al. In vitro inhibition and intracellular enhancement of lysosomal alpha-galactosidase A activity in Fabry lymphoblasts by 1-deoxygalactonojirimycin and its derivatives. Eur J Biochem. 2000;267(13):4179-86. 10.1046/j.1432-1327.2000.01457.x.
4. Fan J.Q., Ishii S., Asano N., Suzuki Y. Accelerated transport and maturation of lysosomal alpha-galactosidase A in Fabry lymphoblasts by an enzyme inhibitor. Nat Med. 1999;5(1):112-5. 10.1038/4801.
5. Lukas J., Cimmaruta C., Liguori L., et al. Assessment of gene variant amenability for pharmacological chaperone therapy with 1-deoxygalactonojirimycin in Fabry disease. Int J Mol Sci. 2020;21(3). 10.3390/ijms21030956.
6. Sugawara K., Ohno K., Saito S., Sakurab H. Structural characterization of mutant alpha-galactosidases causing Fabry disease. J Hum Genet. 2008;53(9):812-24. 10.1007/s10038-008-0316-9.
7. Wu X, Katz E., Della Valle M.C., et al. A pharmacogenetic approach to identify mutant forms of α-galactosidase A that respond to a pharmacological chaperone for Fabry disease. Hum Mutat. 2011;32(8):965-77. 10.1002/humu.21530.
8. Benjamin E.R., Flanagan J.J., Schilling A., et al. The pharmacological chaperone 1-deoxygalactonojirimycin increases alpha-galactosidase A levels in Fabry patient cell lines. J Inherit Metab Dis. 2009;32(3):424-40. 10.1007/s10545-009-1077-0.
9. Lukas J., Giese A.K., Markoff A., et al. Functional characterisation of alpha-galactosidase a mutations as a basis for a new classification system in Fabry disease. PLoS Genet. 2013;9(8):e1003632. 10.1371/journal.pgen.1003632.
10. Porto C., Pisani A., Rosa M., et al. Synergy between the pharmacological chaperone 1-deoxygalactonojirimycin and the human recombinant alpha-galactosidase A in cultured fibroblasts from patients with Fabry disease. J Inherit Metab Dis. 2012;35(3):513-20. 10.1007/s10545-011-9424-3.
11. Seemann S., Ernst M., Cimmaruta C., et al. Proteostasis regulators modulate proteasomal activity and gene expression to attenuate multiple phenotypes in Fabry disease. Biochem J. 2020;477(2):359-80. 10.1042/bcj20190513.
12. Yu Y, Mena-Barragá T., Higaki K., et al. Molecular basis of 1-deoxygalactonojirimycin arylthiourea binding to human α-galactosidase a: pharmacological chaperoning efficacy on Fabry disease mutants. ACS Chem Biol. 2014;9(7):1460-9. 10.1021/cb500143h.
13. Dasgupta N., Xu Y.H., Li R., et al. Neuronopathic Gaucher disease: dysregulated mRNAs and miRNAs in brain pathogenesis and effects of pharmacologic chaperone treatment in a mouse model. Hum Mol Genet. 2015;24(24):7031-48. 10.1093/hmg/ddv404.
14. Khanna R., Benjamin E.R., Pellegrino L., et al. The pharmacological chaperone isofagomine increases the activity of the Gaucher disease L444P mutant form of beta-glucosidase. Febs J. 2010;277(7):1618-38. 10.1111/j.1742-4658.2010.07588.x.
15. Sun Y., Liou B., Xu Y.H., et al. Ex vivo and in vivo effects of isofagomine on acid β-glucosidase variants and substrate levels in Gaucher disease. J Biol Chem. 2012;287(6):4275-87. 10.1074/jbc.M111.280016.
16. Mena-Barragán T., García-Moreno M.I., Sevšek A., et al. Probing the inhibitor versus chaperone properties of sp^2-Iminosugars towards human β-glucocerebrosidase: A picomolar chaperone for Gaucher disease. Molecules. 2018;23(4). 10.3390/molecules23040927.
17. Fog C.K., Zago P., Malini E., et al. The heat shock protein amplifier arimoclomol improves refolding, maturation and lysosomal activity of glucocerebrosidase. eBio Med. 2018;38:142-53. 10.1016/j.ebiom.2018.11.037.
18. Sawkar A.R., Cheng W.C., Beutler E., et al. Chemical chaperones increase the cellular activity of N370S beta -glucosidase: a therapeutic strategy for Gaucher disease. Proc Natl Acad Sci. 2002;99(24):15428-33. 10.1073/pnas.192582899.

19. Sawkar A.R., Adamski-Werner S.L., Cheng W.C., et al. Gaucher disease-associated glucocerebrosidases show mutation-dependent chemical chaperoning profiles. Chem Biol. 2005;12(11):1235-44. 10.1016/j.chembiol.2005.09.007.
20. Sawkar A.R., Schmitz M., Zimmer K.P., et al. Chemical chaperones and permissive temperatures alter localization of Gaucher disease associated glucocerebrosidase variants. ACS Chem Biol. 2006;1(4):235-51. 10.1021/cb600187q.
21. Bendikov-Bar I., Maor G., Filocamo M., Horowitz M. Ambroxol as a pharmacological chaperone for mutant glucocerebrosidase. Blood Cells Mol Dis. 2013;50(2):141-5. 10.1016/j.bcmd.2012.10.007.
22. Maegawa G.H., Tropak M.B., Buttner J.D., et al. Identification and characterization of ambroxol as an enzyme enhancement agent for Gaucher disease. J Biol Chem. 2009;284(35):23502-16. 10.1074/jbc.M109.012393.
23. Aflaki E., Borger D.K., Moaven N., et al. A new glucocerebrosidase chaperone reduces α-synuclein and glycolipid levels in iPSC-derived dopaminergic neurons from patients with Gaucher disease and Parkinsonism. J Neurosci. 2016;36(28):7441-52. 10.1523/jneurosci.0636-16.2016.
24. Flanagan J.J., Rossi B., Tang K., et al. The pharmacological chaperone 1-deoxynojirimycin increases the activity and lysosomal trafficking of multiple mutant forms of acid alpha-glucosidase. Hum Mutat. 2009;30(12):1683-92. 10.1002/humu.21121.
25. Okumiya T, Kroos MA, Vliet LV, Takeuchi H, Van der Ploeg AT, Reuser AJ. Chemical chaperones improve transport and enhance stability of mutant alpha-glucosidases in glycogen storage disease type II. Mol Genet Metab. 2007;90(1):49-57. 10.1016/j.ymgme.2006.09.010.
26. Porto C., Cardone M., Fontana F., et al. The pharmacological chaperone N-butyldeoxynojirimycin enhances enzyme replacement therapy in Pompe disease fibroblasts. Mol Ther. 2009;17(6):964-71. 10.1038/mt.2009.53.
27. Parenti G., Zuppaldi A., Gabriela Pittis M., et al. Pharmacological enhancement of mutated alpha-glucosidase activity in fibroblasts from patients with Pompe disease. Mol Ther. 2007;15(3):508-14. 10.1038/sj.mt.6300074.
28. Matsuda J., Suzuki O., Oshima A., et al. Chemical chaperone therapy for brain pathology in G(M1)-gangliosidosis. Proc Natl Acad Sci 2003;100(26):15912-7. 10.1073/pnas.2536657100.
29. Suzuki Y., Ichinomiya S., Kurosawa M., et al. Chemical chaperone therapy: clinical effect in murine G(M1)-gangliosidosis. Ann Neurol. 2007;62(6):671-5. 10.1002/ana.21284.
30. Maegawa G. H., Tropak M., Buttner J., et al. Pyrimethamine as a potential pharmacological chaperone for late-onset forms of GM2 gangliosidosis. J Biol Chem. 2007;282(12):9150-61. 10.1074/jbc.M609304200.
31. Cheng W.C., Lin C.K., Li H.Y., et al. A combinatorial approach towards the synthesis of non-hydrolysable triazole-iduronic acid hybrid inhibitors of human α-l-iduronidase: discovery of enzyme stabilizers for the potential treatment of MPSI. Chem Commun. 2018;54(21):2647-50. 10.1039/c7cc09642a.
